# Structural and functional insights into the molecular mechanism of rRNA m6A methyltransferase RlmJ

**DOI:** 10.1093/nar/gkt719

**Published:** 2013-08-13

**Authors:** Avinash S. Punekar, Josefine Liljeruhm, Tyson R. Shepherd, Anthony C. Forster, Maria Selmer

**Affiliations:** Department of Cell and Molecular Biology, Uppsala University, PO Box 596, SE 751 24 Uppsala, Sweden

## Abstract

RlmJ catalyzes the m^6^A2030 methylation of 23S rRNA during ribosome biogenesis in *Escherichia coli*. Here, we present crystal structures of RlmJ in apo form, in complex with the cofactor S-adenosyl-methionine and in complex with S-adenosyl-homocysteine plus the substrate analogue adenosine monophosphate (AMP). RlmJ displays a variant of the Rossmann-like methyltransferase (MTase) fold with an inserted helical subdomain. Binding of cofactor and substrate induces a large shift of the N-terminal motif X tail to make it cover the cofactor binding site and trigger active-site changes in motifs IV and VIII. Adenosine monophosphate binds in a partly accommodated state with the target N6 atom 7 Å away from the sulphur of AdoHcy. The active site of RlmJ with motif IV sequence _164_DPPY_167_ is more similar to DNA m^6^A MTases than to RNA m^6^_2_A MTases, and structural comparison suggests that RlmJ binds its substrate base similarly to DNA MTases T4Dam and M.*Taq*I. RlmJ methylates *in vitro* transcribed 23S rRNA, as well as a minimal substrate corresponding to helix 72, demonstrating independence of previous modifications and tertiary interactions in the RNA substrate. RlmJ displays specificity for adenosine, and mutagenesis experiments demonstrate the critical roles of residues Y4, H6, K18 and D164 in methyl transfer.

## INTRODUCTION

Nucleotide methylation is the most frequent type of post-transcriptional modification of rRNA in *Escherichia coli* ribosomes (1)*.* Recently, the *yhiR* gene product (Uniprot ID: P37634) was identified as the site-specific methyltransferase (MTase) enzyme responsible for S-adenosyl-methionine (AdoMet)-dependent monomethylation of the exocyclic N6 atom of adenosine (m^6^A) at nucleotide 2030 in *E. coli* 23S rRNA and was consequently renamed RlmJ (2).

The 36 rRNA modifications in *E. coli* cluster around the functional centers of the ribosome: the decoding center, the transfer RNA (tRNA) binding A and P sites, the peptidyl transferase center (PTC) and the peptide exit tunnel. However, none of the rRNA modification enzymes in *E. coli* have been shown to be essential for viability in individual knockouts. The specific roles of many rRNA modifications remain unknown, and the current understanding is that they, to a large extent, have evolved in a conserted way to fine-tune the structure and function of the ribosome [reviewed in (1)].

**Figure 1.**
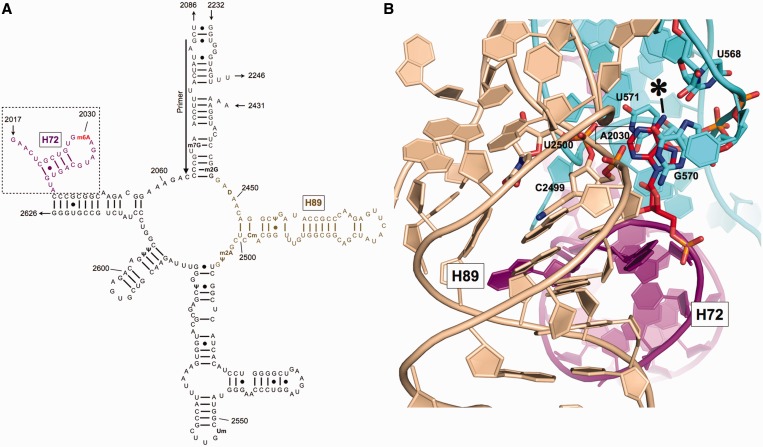
Location of A2030 in *E. coli* 23S rRNA. (**A**) Secondary structure near the central loop region of domain V of *E. coli* 23S rRNA [based on PDB 2QAM (3)]. m^6^A2030 is shown in red and helices 72 and 89 are in magenta and wheat color, respectively. An arrow indicates the primer-binding site for extension analysis. A dotted box indicates the minimal hairpin fragment of H72 on which RlmJ shows MTase activity. (**B**) The 3D structure surrounding A2030 in the *E. coli* 70S ribosome [PDB 2QAM (3)]. N6 of A2030 is indicated by an asterisk, H72 is shown in magenta, helix 89 in wheat and the G570 region of domain II in cyan. Residues that make contact with A2030 are labeled.

Nucleotide A2030, the modification site of RlmJ, is located in the hairpin loop of helix 72 (H72) at the 5′ boundary of domain V in 23S rRNA (Figure 1A). In the mature 50S (3), this loop is involved in tertiary interactions where m^6^A2030 stacks between G570 and U571, with the N6 closest to the 2′O and O2 of U568 in domain II, whereas the following base, A2031, stacks between C961 in domain II and C2498 in the PTC region of domain V (Figure 1B). The modification is hidden in the interior of the subunit, agreeing with its appearance at an early stage of 50S assembly (4) and with the observation that RlmJ specifically methylates deproteinized knockout 23S rRNA, but not assembled 50S subunits (2).

In *E. coli*, two additional enzymes methylate rRNA adenosines at the N6 positions. These are RlmF (YbiN) that monomethylates adenosine 1618 in 23S rRNA (5) and KsgA (RsmA) that N6,N6-dimethylates adenosines 1518 and 1519 in 16S rRNA (6). Although KsgA has been extensively studied (6–8), no structural information is available for the enzymes RlmF and RlmJ.

In addition to rRNA, m^6^A modifications also occur in tRNA, mRNA, small nucleolar RNA and non-coding RNA as well as in DNA. RlmJ was found to contain a sequence motif characteristic for m^6^A MTases acting on DNA (9) and has also been implicated in the ability of bacteria to use DNA as nutrient (9) and in repression of plasmid uptake (10), but these processes are poorly understood. The knockout of *rlmJ* does not affect the growth rate (2) but lowers the competitive fitness at long-term growth in stationary phase (9) and provides a small growth advantage under anaerobic conditions (2).

Here, we present the structure of RlmJ, demonstrate its substrate requirements and specificity and identify functionally critical residues in its active site.

## MATERIALS AND METHODS

### Crystallization and crystallographic data collection

We have previously reported the cloning, expression, purification, crystallization and data collection of RlmJ_APO_ with a C-terminal hexa-histidine tag (11). A complex of RlmJ (11 mg/ml) with S-adenosyl-methionine (AdoMet, 1.0 mM), i.e. RlmJ_SAM_ was crystallized under identical conditions after streak seeding from an apo RlmJ crystal. To obtain a complex of RlmJ with S-adenosyl-homocysteine (AdoHcy) and adenosine monophosphate (AMP), i.e. RlmJ_SAH-AMP_, RlmJ_APO_ crystals were soaked in mother liquor containing 5 mM AdoHcy and 20 mM adenosine triphosphate (ATP) for 15 min before cryoprotection. All X-ray diffraction data were collected at 100 K, processed with XDS (12) and scaled with XSCALE (12). Data statistics are summarized in Table 1.

**Table 1.** Summary of crystallographic data and refinement statistics

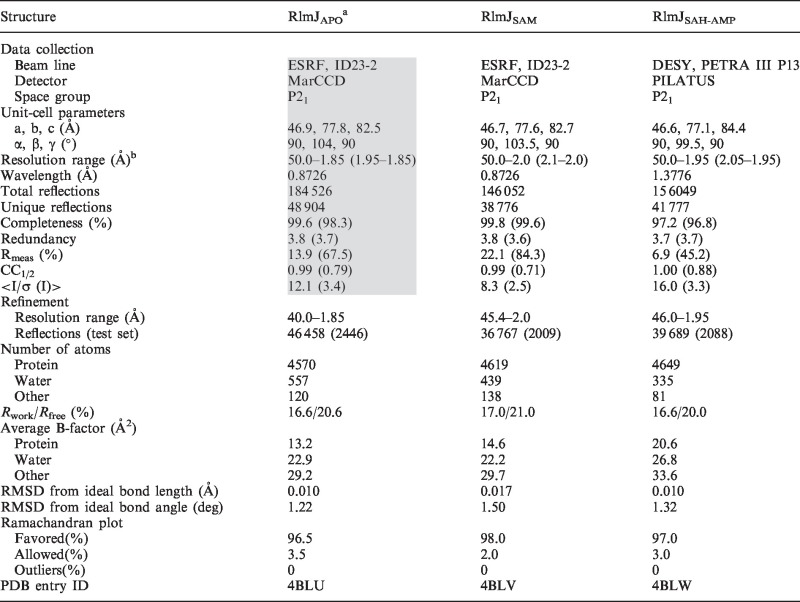

^a^Data statistics in shaded area are reported in (11)

^b^Values within parentheses represent the highest resolution bin.

### Structure determination

Molecular replacement (MR) was performed using Phaser (13) and model building using Coot (14). Ligand coordinates and CIF restraint definitions of AdoMet, AdoHcy and AMP were obtained using JLigand (15). Refinement was done in PHENIX (16). The quality of the refined structures was assessed using MolProbity (17).

The RlmJ_APO_ structure was solved by MR using protein data bank (PDB) entry 2OO3 edited using Sculptor (18) as a search model. Two molecules were located in the asymmetric unit. The resulting model was subjected to manual rebuilding and refined to 1.85 Å resolution.

The RlmJ_SAM_ structure was solved by rigid-body refinement of the RlmJ_APO_ structure against the RlmJ_SAM_ data, and the RlmJ_SAH-AMP_ structure was solved by MR using the RlmJ_APO_ structure as search model. After refinement of the polypeptides, the ligands AdoMet, AdoHcy and AMP (only one phosphate of ATP visible) were fitted into the respective F_o_-F_c_ electron density maps. The structures of RlmJ_SAM_ and RlmJ_SAH-AMP_ were refined to 2.0 and 1.95 Å resolution, respectively.

Refinement statistics are presented in Table 1. The structure factors and refined coordinates have been deposited in the PDB. Structure figures were prepared using PyMOL (The PyMOL Molecular Graphics System, Version 1.5, Schrödinger, LLC).

### Sequence analysis, surface mapping of conserved residues and electrostatic surface potential

NCBI position-specific iterative (PSI) BLAST (19) was used to search for RlmJ homologs from the non-redundant protein sequence database. Multiple sequence alignment was done using Clustal Omega (20). The alignment figure was generated using ESPript (21). Protein charge distribution was calculated using the PARSE force field in PDB2PQR (22), and electrostatic surface potential maps were generated using APBS (23) in PyMOL.

### *In vitro* transcription and RNA preparation

23S rRNA *in vitro* transcript [IVT; (24)] was synthesized by T7 RNA polymerase using pCW1 plasmid DNA template (25) cut with AflII (Fermentas). H72 RNA with an additional 5′-GG sequence was synthesized by T7 RNA polymerase from a DNA template of polyacrylamide gel purified oligonucleotides (IDT) containing the wild-type A or the T, C and G point mutations at the 2030-equivalent position (Supplementary Table S1). The resulting transcription product was treated with DNase I (Fermentas) for 1 h at 37°C and purified from a 12% polyacrylamide gel. Forward DNA corresponding to the wild-type H72 sequence was also synthesized (Supplementary Table S1).

The 23S rRNA from the wild-type BW25113 strain (26) was extracted using sodium dodecyl sulfate/phenol extraction and ethanol precipitation from cells grown in Luria-Bertani medium (27) at 37°C overnight.

### *In vitro* modification and primer extension analysis

IVT was heated at 50°C for 3 min followed by 37°C for 10 min. Then, 200 μM AdoMet was added as the methyl donor to 11 pmol IVT in 50 mM HEPES–KOH (pH 7.5), 100 mM NaCl, 5 mM β-mercaptoethanol, 1 mM Mg(OAc)_2_ (reaction buffer), followed by addition of 11 pmol purified RlmJ and bringing to 50 μl total. The reactions proceeded at 37°C for 30 s or 30 min, as indicated, and were quenched by 50:50 phenol:chloroform extraction followed by ethanol precipitation. RNA was dissolved in H_2_O before primer extension.

Primer extension analysis was carried out as described previously (24), with the exception that the 5′-^32^P-labeled primer was complimentary to the *E. coli* 23S rRNA nucleotide sequence 2063–2083 (5′-GAUAUCAUUUCCAAGUGCCCC-3′; IDT). Additionally, deoxynucleotide concentrations were optimized to 100 μM deoxy-GTP, 100 μM deoxy-CTP, 100 μM deoxy-ATP and 10 μM deoxy-TTP to best visualize differences between unmodified and modified rRNA on primer extension.

### Site-directed mutagenesis

Six mutants of RlmJ (Y4A, Y4F, H6D, K18A, K18R and D164A) were constructed by site-directed mutagenesis using the QuikChange II protocol (Stratagene). Briefly, mutations were introduced by PCR using the plasmid pAP01-rlmJ (11) as template in combination with mutagenic primers (Supplementary Table S1) and verified by DNA sequencing. RlmJ mutants were expressed and purified as described previously (11).

### *In vitro* modification and tritium labeling analysis

In all, 40 pmol IVT, transcribed H72 RNA and H72 RNA mutants or single-stranded DNA having the same sequence as wild-type H72 were resuspended in reaction buffer and heated for 3 min at 50°C followed by 10 min at 37°C. Addition of 400 pmol unlabeled AdoMet (Sigma-Aldrich, USA) doped with 4 pmol S-[methyl-^3^H]-adenosyl-l-methionine (^3^H-AdoMet) (4 Ci/mmol; PerkinElmer, USA) was followed by the addition of 20 pmol wild-type or mutant RlmJ, or buffer alone and brought to 50 μl total. All reactions were carried out at 37°C for 30 min and quenched in 2 ml of ice cold 10% trichloroacetic acid and incubating on ice for 10 min. The precipitations were applied to a BA85 nitrocellulose filter (Whatman, UK) under vacuum and washed five times with 7 ml of 10% cold trichloroacetic acid. The washed filters were then placed in vials containing 5 ml of Filter Safe scintillation cocktail (Shleicher & Schuell, Germany), shaken for 30 min and counted in an LC6500 scintillation counter (Beckman, USA). Expected counts per minute (cpm) of quantitative labeling with 40 pmol ^3^H based on the specific activity and quenching titrations were 14 100 cpm. Notably, all assays were done within 1 day of enzyme purifications, as the enzyme is unstable when stored over time.

## RESULTS

### Structure determination of RlmJ

The 1.85 Å RlmJ_APO_ structure was solved by MR using PDB entry 2OO3, hypothetical protein LPL1258 from *Legionella pneumophila,* as a search model. A BLAST search of the PDB revealed that this crystal structure, annotated as a protein involved in catabolism of external DNA, displayed 37% sequence identity to *E. coli* RlmJ. Thus, LPL1258, for which there is no published function, most likely represents an RlmJ homologue. The RlmJ structure was solved in space group P2_1_ with two monomers in the asymmetric unit related by a 2-fold non-crystallographic symmetry. The structure suggests that RlmJ functions as a monomer, consistent with its behavior in size exclusion chromatography (11). The final models consist of residues 2–280, but with residues 53–55 missing in the second molecule.

### Overall structure

**Figure 2.**
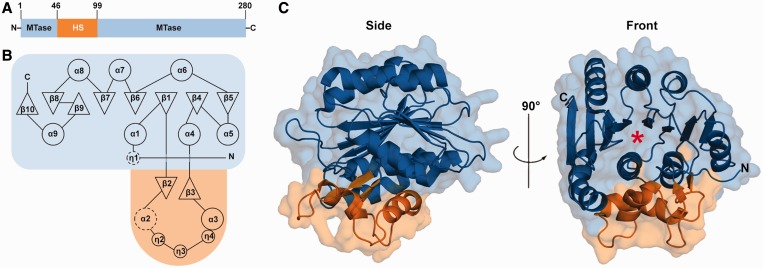
Overall structure of *E. coli* RlmJ. The MTase domain is shown in blue and the HS in orange. (**A**) Domain organization of RlmJ. (**B**) Topology diagram. β-strands are shown as triangles, α-helices are shown as large circles, and 3_10_-helices are shown as small circles. Dotted circles indicate helices formed on binding of cofactor and substrate (see Figure 4B). (**C**) Cartoon representation shown in side and front view. A red asterisk indicates the substrate binding site.

The RlmJ structure consists of a discontinuous MTase domain (residues 1–46 and 99–280), interrupted by a helical subdomain (HS, residues 47–98) (Figure 2A and B). Together, they form a compact and globular 40.5 × 37.5 × 47.5 Å^3^ structure with a prominent pocket on one side (Figure 2C). The MTase domain of RlmJ consists of a central twisted eight-stranded β-sheet flanked by three α-helices on one side and four α-helices on the other side. The first six strands of the β-sheet are parallel, and the last two strands are antiparallel. An extra helix α9 and strand β10 at the C-terminal end of RlmJ distinguishes it from the canonical class I Rossmann-like MTase fold (28).

**Figure 3.**
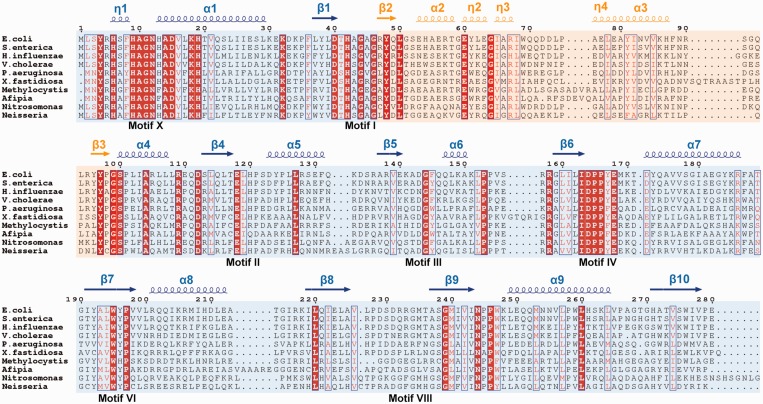
Sequence alignment of a representative set of RlmJ sequences from proteobacteria. Conserved residues are shown in white on red background, and conservative substitutions are in red. The blue background indicates the MTase domain, and the orange background indicates the HS. Secondary structure of *E. coli* RlmJ is indicated above the alignment, and sequence motifs of DNA m^6^A MTases (29) are indicated below the alignment. NCBI accession numbers of the sequences are as follows: *Escherichia coli* (NP_417956.1), *Salmonella enterica* (YP_001590613.1), *Haemophilus influenzae* (YP_004136235.1), *Vibrio cholerae* (ZP_17776694.1), *Pseudomonas aeruginosa* (ZP_15628770.1), *Xylella fastidiosa* (NP_297336.1), *Methylocystis sp.* (YP_006593692.1), *Afipia sp.* (ZP_07027437.1), *Nitrosomonas sp.* (YP_004696048.1) and *Neisseria sp.* (ZP_06980365.1). The figure was generated using ESPript (21).

The location of the inserted subdomain (Figures 2 and 3A) is novel among AdoMet-dependent MTases (28). The strands β2 and β3 form a hairpin into which the three 3_10_-helices and two α-helices are inserted. Residues 53–58 show weak electron density and form helix α2 only in the closed conformation of the N-terminus (see later in the text). The interface between the HS and the MTase domain is mainly hydrophobic.

A search for similar structures using the DALI server (30) showed that only PDB entry 2OO3, which was used as search model in MR, displays significant similarity to the full RlmJ structure (*Z*-score 30). RlmJ displays lower structural similarity to many other MTase enzymes that modify a variety of small molecule, nucleic acid and protein substrates (Supplementary Table S2). Hits with *Z*-scores of 12–13 and low sequence identity (7–18%) align with the core of the MTase domain and include human MTase 10 domain containing protein (hMT10, PDB 2H00, unpublished), (N5)-glutamine MTase HemK [PDB 1T43, Z. (31)], catechol O-MTase [PDB 1VID, (32)], tRNA m(2)G6 MTase TrmN [PDB 3TMA, (33)], rRNA m2G966 MTase RsmD [PDB 2FPO, (34)] and an archaeal ortholog of rRNA m2G1207 MTase RsmC [PDB 1DUS, (35)].

Of these, only hMT10 is likely to represent an m^6^A-specific MTase, as it displays >25% sequence identity to *E. coli* RlmF, responsible for the 23S m^6^A1618 modification (5). Structures of DNA m^6^A MTases (M.*Taq*I, DpnM, T4Dam, *Eco*Dam) and RNA m^6^_2_A MTases (KsgA, Dim1, ErmAM and ErmC') displayed *Z*-scores below 11. A DALI search with only the HS did not produce any significant hits.

### Multiple sequence alignment

A PSI-BLAST search identified full-length homologs of *E. coli* RlmJ in eubacteria, the majority in proteobacteria and few in spirochaetes and verrucomicrobia. Multiple sequence alignment of *E. coli* RlmJ and a representative set of homologs (Figure 3A) revealed a cluster of 10 strictly conserved residues among the 19 first amino acids, suggesting an important role of the N-terminus. The remaining 25 conserved residues are spread throughout the sequence.

Comparison of the RlmJ structure with hits from the DALI search allowed localization of seven of the nine conserved sequence motifs (I–VIII and X) that are specifically arranged in AdoMet-dependent DNA MTases (29), which could be mapped in the sequence alignment (Figure 3). Of these, the conserved N-terminus (residues 1–19) constitutes motif X and DPP(Y/F) (residues 164–167) is motif IV within the sequence 161–167 that matches the [LIVMAC]-[LIVFYWA]-{DYP}-[DN]-P-P-[FYW] PROSITE DNA m^6^A MTase consensus pattern. Interestingly, the sequence alignment also shows a second conserved match (amino acid 241–247) to the same PROSITE signature that is located at the opposite side of the structure and has no equivalent in the other MTases.

### Complexes of RlmJ with cofactor and substrate analogue

**Figure 4.**
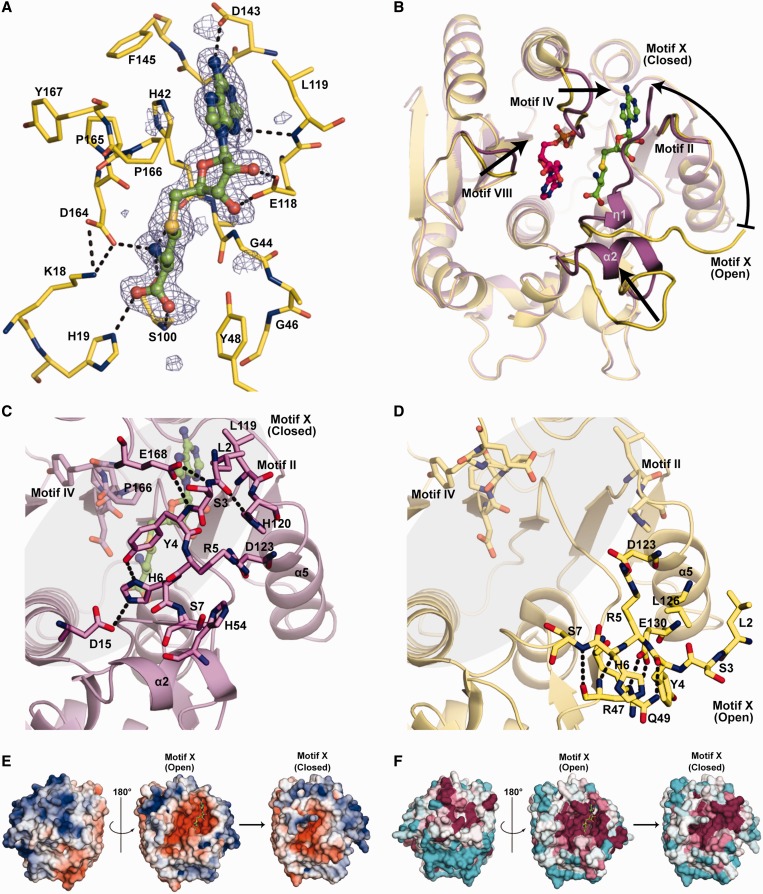
(**A**) The cofactor binding site in RlmJ. The unbiased F_o_-F_c_ map (blue) of AdoHcy is contoured at 2.2σ (0.3503 e^-^/Å^3^). AdoHcy is in green, the interacting residues within hydrogen bonding distance are shown in yellow and hydrogen bonds as dotted lines. (**B**) Comparison of the RlmJ_APO_ (pale yellow) and the RlmJ_SAH-AMP_ complex (purple). Binding of AdoHcy (green) and the substrate AMP (pink) to RlmJ triggers structural changes as indicated by the arrows. (**C**) Close-up view of motif X in closed conformation and (**D**) in open conformation. The active site in RlmJ is highlighted in gray. AdoHcy is shown in green. Interacting side chains are shown as sticks and hydrogen bonds as dotted lines. (**E**) Electrostatic surface potential of RlmJ_SAM_. The color spectrum ranges from deep red (–5 kT) to deep blue (+5 kT). (**F**) Surface representation of RlmJ_SAM_ colored according to sequence conservation using ConSurf (36). The color spectrum ranges from magenta (highest conservation) to cyan (lowest conservation). Orientation of the middle view (motif X open) as in Figure 4B. AdoMet is shown in green. The closed form of the motif X tail completely buries AdoMet.

Structures of RlmJ in a binary complex with the methyl donor AdoMet (RlmJ_SAM_) and in a ternary complex with the cofactor reaction product S-adenosyl-homocysteine (AdoHcy) and the substrate analogue AMP (RlmJ_SAH-AMP_) were obtained through co-crystallization and soaking, respectively. The unbiased F_o_-F_c_ difference electron density maps of RlmJ_SAM_ and RlmJ_SAH-AMP_ showed ordered binding of AdoMet/AdoHcy (Figure 4A) and of the adenosine and ribose moieties of AMP (Supplementary Figure S1) in each RlmJ monomer. Weak density was observed for the α-phosphate of AMP.

Comparison of the structures shows that one molecule in the RlmJ_SAM_ structure has the same conformation as the RlmJ_APO_ structure, whereas the other molecule takes the conformation of the RlmJ_SAH-AMP_ structure [root mean square deviation (RMSD) of 0.2 Å over 280 C_α_ atoms for the respective comparison]. The RlmJ_SAH-AMP_ structure shows conformational changes in four loop regions compared with RlmJ_APO_ (Figure 4B). The N-terminal tail (residues 1–8 of motif X) performs a striking 88° rotation around the α1 helical axis from an open conformation in RlmJ_APO_ to a closed conformation in RlmJ_SAH-AMP_ resulting in a 19 Å shift of the C_α_ of residue L2 and formation of a short 3_10_ helix η1, loop residues 53–58 in the HS forms a helix α2, residues 165–170 in and after motif IV are rearranged to make contact with the N-terminal tail and constrict the cofactor-binding pocket, and residues 232–234 in motif VIII are rearranged to constrict the substrate-binding pocket (Supplementary Figure S2, see later in the text).

In the closed form, motif X becomes a part of the active site. This conformation is mainly stabilized by the motif II loop (residues L119–D123) from one side and the motif IV loop (P166 and E168) and helix α2 from the other side (Figure 4C). These interactions also cause a motif II constriction toward the active site. In the open form, motif X does not approach the active site, and instead is stabilized by interactions with strand β2 (R47 and Q49) and helix α5 (residues D123–E130) (Figure 4D).

### Cofactor binding site

The conserved residues in RlmJ mainly cluster in and around a deep, negatively charged, L-shaped pocket. AdoMet binds in an extended conformation to one part of the pocket and is completely buried by the motif X tail in closed conformation (Figure 4E and F). The adenine and the ribose of the cofactor are surrounded by conserved residues in motifs I, II, III and IV (Supplementary Figure S3A, Figure 3). The adenine is sandwiched between H42 of motif I and L119 of motif II. H42 and F145 of motif III form a hydrophobic bottom of the adenine-binding site. The adenine is further stabilized by hydrogen bonds to the side chain of D143 and the backbone amide of G144 in motif III. The ribose is positioned by hydrogen bonds to the E118 side chain of motif II. The amino acid moiety enters into a pocket, where it hydrogen bonds to S100 and D164. Additionally, hydrogen bonds from the backbone carbonyl of H42 and the H19 side chain in motif X stabilize its carboxyl and amide groups. The interaction between motif X and the cofactor has not been observed in other MTases.

In the closed conformation, the side chains of motif X form an intricate network of interactions: the imidazole ring of H6 forms hydrogen bonds to Y4 and D15 that position Y4 and H6 between the cofactor and the substrate binding site (Figure 4C). D15 also positions K18 through a hydrogen bond, as discussed later in the text.

### Substrate binding site

In the RlmJ_SAH-AMP_ structure, AMP is bound in the substrate binding pocket, surrounded by several water molecules. The substrate interacts with conserved residues from the motif IV region, motif VI and motif X of the MTase domain (see later in the text) and residue E60 from the HS. Additionally, non-conserved residues from the motif VIII loop contribute. Residues A14, Y167, W195, P197, V199 and M235 provide a hydrophobic surface on one side of the pocket. The adenine base is positioned by hydrogen bonds from the hydroxyl group of Y4 and the amide group of N12, and by interaction with H9 on one side and M235 on the opposite side (Supplementary Figure S3B).

The adenine base is not inserted far enough into the pocket for catalysis. The exocyclic N6 is at a distance of 7 Å from the sulphur atom of AdoHcy, whereas the maximum distance between these two atoms in a direct methyl transfer mechanism would be ∼5 Å (37). The N6 is also outside hydrogen bonding distance from the proton acceptors in motif IV (see later in the text).

RlmJ displays a positively charged surface that could function in binding of the rRNA substrate (Figure 4E). However, this surface is not very conserved (Figure 4F).

### The catalytic site of RlmJ is similar to m^6^A DNA MTases

The structure of RlmJ is the first one of an enzyme with demonstrated monomethylating m^6^A RNA MTase activity. The motif IV sequence (_164_DPPY/F_167_, Figure 3) agrees with the (N/D)PP(Y/F/W) motif of monomethylating DNA m^6^A MTases such as DpnM, *Eco*Dam, T4Dam and M.*Taq*I and not with the (A/S/N)(L/I/V)P(Y/F) motif of dimethylating RNA m^6^_2_A MTases KsgA and ErmC′ as defined in the NCBI conserved domain database (38). Also, the structure of motif IV in RlmJ is similar to a DNA m^6^A MTase (Supplementary Figure S4). In RNA m^6^_2_A MTases, the small hydrophobic residue that replaces the first proline induces changes in the backbone that sterically allow re-binding and methylation of previously monomethylated substrates (39,40). Thus, motif IV in RlmJ is characteristic for an m^6^A MTase that transfers one methyl group and motivates further comparison with such enzymes acting on DNA substrates.

### Structural model of a catalytic complex

**Figure 5.**
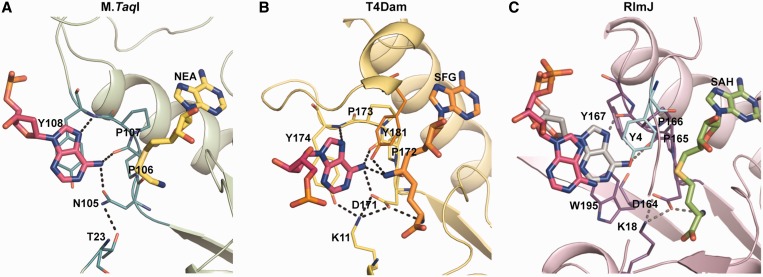
Binding of substrate-adenosine (pink) to the active sites of m^6^A DNA MTases and RlmJ. Hydrogen bonds are shown as dotted lines. (**A**) M.*Taq*I-DNA complex with the cofactor analogue 5′-deoxy-5′-[2-(amino)ethylthio]adenosine [NEA, PDB 1G38 (37)] (**B**) T4Dam-DNA complex with the cofactor analogue sinefungin [SFG, PDB 1YFL (41)]. (**C**) RlmJ_SAH-AMP_ with the modeled substrate adenosine (gray) positioned according to superpositioning with M.*Taq*I.

Superposition of structures of DNA m^6^A MTases in complex with cofactor-like inhibitors and DNA substrate onto RlmJ_SAH-AMP_ provides us with a model for binding of accommodated substrate adenosine to RlmJ (Figure 5). The T4Dam complex [PDB 1YFL, (41)] and the M.*Taq*I complex [PDB 1G38, (37)] superpose on RlmJ_SAH-AMP_ with RMSDs of 2.1 Å over 113 C_α_ atoms and 2.0 Å over 107 C_α_ atoms, respectively, and the cofactor analogues overlay well.

In T4Dam, a helix in the region after motif IV covers the cofactor analogue and positions Y181 between the target adenine and the atom equivalent to the sulphur atom of AdoMet. Interestingly, the first four residues in motif X of RlmJ superpose onto this helix of T4Dam, and Y4 of RlmJ has identical orientation toward the adenosine N6 as Y181 in T4Dam (Figure 5B).

The substrate base in both DNA MTases is stabilized by interactions with the D/NPPY motif. N6 of the substrate base is hydrogen bonded to the catalytic D/N (D171 in T4Dam and N105 in M.*Taq*I) that acts as proton acceptor during catalysis. This residue is positioned by a hydrogen bond to a residue in the motif X tail (K11 in T4Dam and T23 in M.*Taq*I) that directly or indirectly also interacts with N1 of the base. RlmJ has a strictly conserved K18 in helix α1 of motif X that in our model would make a similar interaction with D164 and the base, despite the location of its C_α_ atom at a distance of 6.4 Å from the K11 in T4Dam.

In the DNA MTases, additional hydrogen bonds to the base occur with the backbone carbonyl of the first Pro and the amide of the Tyr. The aromatic ring of the Tyr stacks with the substrate adenine base. In contrast, the side chain of Y167 in motif IV of RlmJ is oriented away from the substrate to engage in hydrophobic interactions. This induces a distinct backbone conformation where the carbonyl groups of both prolines are available for interactions with the substrate. In RlmJ, instead, the conserved W195 of motif VI is ready to stack with the target base and occupies the same space as the motif IV tyrosine in the other structures.

We predict that in the catalytic complex of RlmJ and 23S rRNA, the adenine base is likely to be positioned as in T4Dam and M.*Taq*I (Figure 5C), but the distances from the N6 position to D164 of motif IV and K18 in motif X would be above 4 Å, indicating that a slight movement of motifs IV and X will take place on binding of the correct substrate.

### RlmJ modifies *in vitro* transcribed 23S rRNA at A2030 and requires only H72 for activity

**Figure 6.**
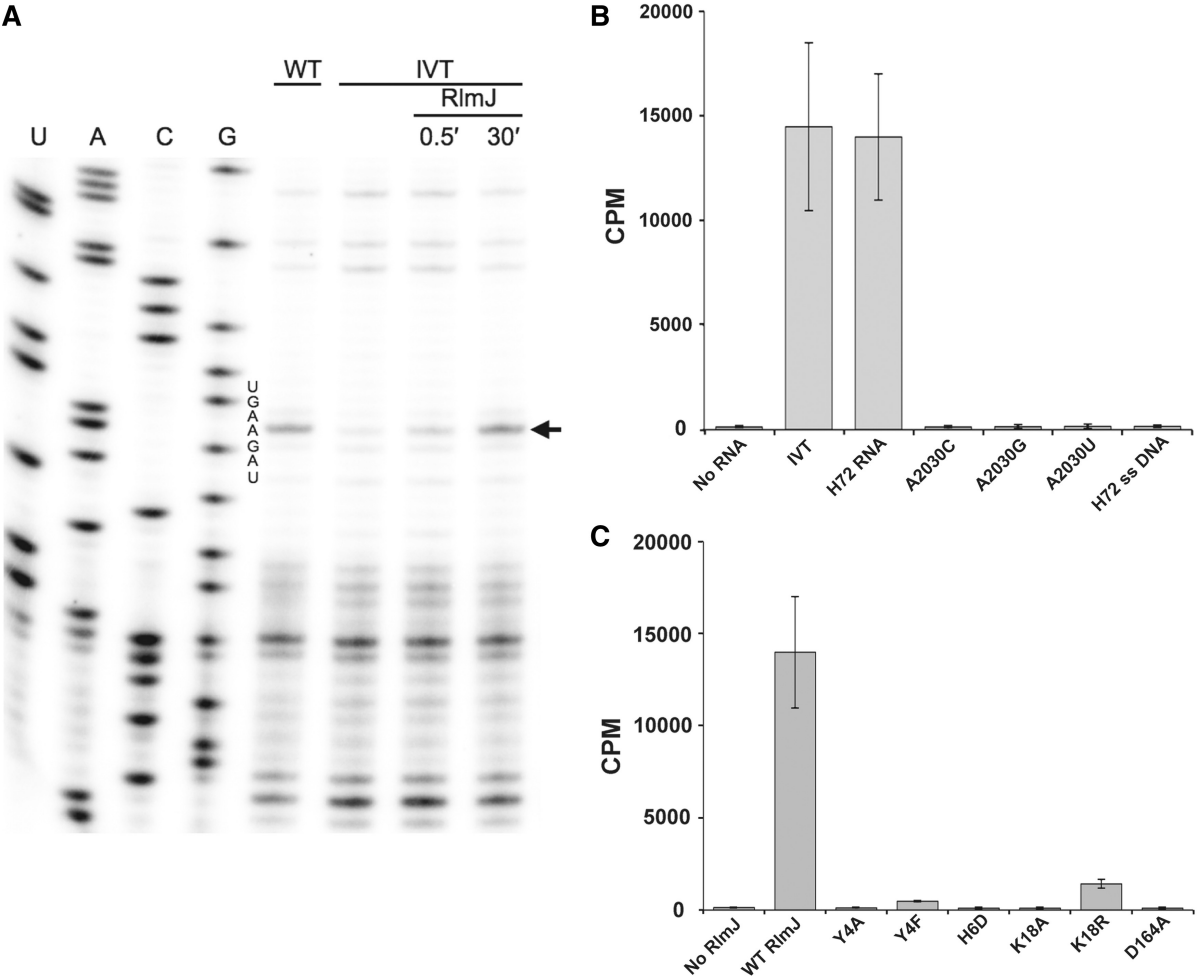
Recombinant RlmJ methylates A2030 in unmodified 23S rRNA transcripts. IVT: *in vitro* transcript of 23S rRNA. (**A**) RlmJ specifically modifies A2030 of IVT based on primer extension. The arrow denotes the extension stop due to the methylation. WT: 23S rRNA purified from a wild-type *E. coli* strain; RlmJ: IVT incubated with the RlmJ enzyme for 30 s (0.5′) and 30 min (30′). (**B** and **C**) Tritium methyl incorporation experiments showing that RlmJ specifically modifies IVT and H72 RNA, requiring strict conservation of the catalytic core for activity. Error bars indicate standard deviations of triplicate measurements. Complete modification was expected to give 14 100 cpm calculated from the quenching titration. Abbreviations are as in text.

Methylation on A2030 in 23S rRNA occurs early in the ribosome biogenesis pathway (2,4), warranting investigation of protein-free unmodified ribosomal RNA as a potential substrate for RlmJ. Indeed, purified recombinant RlmJ modified full-length *in-vitro*-transcribed 23S rRNA (IVT) in the presence of saturating amounts of AdoMet. Following the reaction, primer extension analysis by AMV reverse transcriptase was used to verify the site of modification, mindful that incorporation of complementary deoxyribonucleotides stops at cleavage sites and strong secondary structures in addition to modification sites (42). A prominent stop occurred at A2031 for wild-type 23S rRNA and for IVT incubated with RlmJ, but not IVT alone (Figure 6A). It was concluded that RlmJ modifies A2030 of IVT *in vitro*, with already notable modification by 30 s and attaining near wild-type levels within 30 min.

Deletion analysis revealed that just a single 27 nt helix of the 2904 nt 23S rRNA is sufficient as a substrate. IVT and H72 RNA were incubated with ^3^H-AdoMet and RlmJ, and the amount of ^3^H-methyl labeled RNA was quantified, showing RlmJ methylates both substrates to quantitative levels within 30 min (Figure 6B). To further test substrate specificity, RlmJ was incubated under these same conditions with single-site directed mutants of H72, having A2030 replaced by cytidine (A2030C), guanosine (A2030G) or uridine (A2030U), and, additionally, against single-stranded DNA having the same sequence as wild-type H72 (ss DNA). There was no detectable MTase activity on any of the mutant RNAs or the single-stranded DNA (Figure 6B). These data show RlmJ is highly specific to adenosine at position 2030, requiring only a small hairpin for activity.

### Y4, H6, K18 and D164 of RlmJ are necessary for catalytic activity

Our structural analysis indicated the importance of residues Y4, H6, K18 and D164 for ligand binding and catalysis in RlmJ, which was tested by site-directed mutagenesis. All mutant proteins expressed and purified as wild-type and were tested for MTase activity against H72 RNA.

To test the importance of the phenyl ring and hydroxyl group of Y4, the residue was mutated to Ala (Y4A) and Phe (Y4F). Although catalytic activity of Y4A was completely abrogated, the Y4F mutant maintained some activity, albeit 40-fold less than wild-type RlmJ (Figure 6C). Similar to Y4A, mutating H6 to Asp (H6D) also showed a complete loss of activity (Figure 6C).

To verify that the catalytic residues K18 and D164 are necessary for activity, each residue was individually mutated to Ala (K18A and D164A). Additionally, K18 was mutated to Arg (K18R) to test whether a more subtle change to this highly conserved residue would affect enzyme activity. As expected, mutating either of the residues at the catalytic center to Ala was enough to completely abolish activity (Figure 6C). Furthermore, even the K18R mutant showed a 10-fold loss in activity (Figure 6C). Combined, these data verify the conclusion drawn from the structure on the highly specific coordination of Y4, H6, K18 and D164 for RlmJ activity.

## DISCUSSION

### Structure of RlmJ

The structure of *E. coli* RlmJ shows a unique fold for the RlmJ family, to which the mis-annotated structure of the RlmJ homologue LPL1258 from *L. pneumophila* belongs*.* Many classes of MTases have similar Rossmann-like folds, and additions of extra secondary structure elements at the C-terminus of the core MTase fold are commonly found in MTases targeting larger substrates such as proteins and nucleic acids (28).

Most AdoMet-dependent RNA and DNA MTases in addition to the catalytic domain have a target recognition domain that contributes affinity and specificity to the nucleic acid target (28), which may simplify the process of evolving MTases with new targets. In RlmJ, the inserted HS may contribute to target recognition, but it displays limited surface conservation (Figures 3 and 4F). What is conserved, however, is the interface to the MTase domain and the binding site for the N-terminal motif X in its closed conformation (Figure 4C). This suggests that the insert may have a role in coordination of the open and closed conformations in relation to binding of cofactor and substrate.

### The catalytic site of RlmJ is similar to m^6^A DNA MTases

The catalytic DPP(Y/F) motif IV of RlmJ is identical to the (N/D)PP(Y/F/W) motif of m^6^A DNA MTases. Structural comparison of RlmJ with other N6-adenosine-specific MTases confirms that the catalytic site of RlmJ is more similar to DNA m^6^A MTases than to RNA m^6^_2_A MTases, suggesting that RlmJ uses a similar catalytic mechanism. In the DNA m^6^A MTase enzymes, the catalytic D/N and the main-chain carbonyl oxygen of the first proline in motif IV (Figure 5A and B) deprotonate N6 of the substrate adenosine and activate it for receiving the methyl group from AdoMet in a direct S_N_2 reaction (37,41,43).

In RlmJ, the mutation D164A abolishes all activity (Figure 6C), as previously observed with equivalent mutants in EcoDam (44) and EcoRV (45). Further, absence of activity for the K18A mutant and the lowered activity for the K18R mutant proves the importance of the interactions of K18 with D164 and the substrate base. In EcoRV, the corresponding K16A mutant is also inactive and deficient in AdoMet binding (45).

### Complexes with cofactor and substrate

Complex structures of RlmJ show that the methyl donor AdoMet and the reaction product AdoHcy bind to the canonical cofactor binding site of AdoMet-dependent MTases with Rossmann-like fold, correctly positioned close to the catalytic tetrad _164_DPPY_167_.

In our RlmJ_SAH-AMP_ structure, the target adenosine is located too far away from the catalytic residues and the cofactor. This seems to be a common observation for ternary complexes of related DNA and RNA MTases; with KsgA, there is no structure with a target base in the active site (46), and for T4Dam, the target base is observed in a similar half-inserted state in presence of AdoHcy (41). The presence of cofactor product AdoHcy may signal that the substrate should be released (41). It is also possible that binding of a larger RNA substrate is needed to trigger conformational changes in RlmJ that allow productive binding of the target base.

Inspired by a structure of T4Dam with fully inserted target base solved in presence of the competitive inhibitor sinefungin (41), we solved a structure of RlmJ in complex with the same inhibitor and adenosine (data not shown). While sinefungin bound in the expected position, adenosine bound 2.3 Å further into the active site, but in a non-physiological orientation where the C2 atom instead of N6 pointed toward the cofactor. A similar inward shift of the substrate base would be sufficient to position the N6 close enough to D164 for catalysis.

Comparison with T4Dam and M.*Taq*I allowed us to model the catalytic position of adenosine in RlmJ. In ternary complex structures of these DNA MTases, the distance between the target N6 and the atom mimicking the sulphur of AdoMet is 4 Å (37,41). Although the catalytic motif IV in RlmJ is identical to the motif in DNA m^6^A MTases, the comparison shows that its detailed structure is not conserved. The tyrosine Y167 of the DPPY motif is engaged in hydrophobic interactions rather than stacking with the target base, inducing a different backbone structure and novel interactions with the substrate.

### The critical role of the N-terminal motif X tail

On binding of cofactor and substrate mimic to RlmJ, the N-terminal motif X tail undergoes a large conformational change to enclose the cofactor and contribute to the substrate-binding pocket. The closure of motif X induces movements of motifs II and IV toward the substrate base to form an active site ready for catalysis. The importance of the closed motif X and the interactions of Y4 and H6 for catalysis were verified by mutagenesis. The 40-fold reduced activity of the Y4F mutant proves that the hydrogen bond between Y4 hydroxyl group and H6 is vital for positioning both residues for interaction with the accommodated substrate and the cofactor, which was verified by the abolished activity after Y4A and H6D mutations (Figure 6C). The latter mutation would disrupt the favorable interaction with D15.

Interactions between the helical part of motif X and the cofactor has not been observed in any related protein. In structures of DNA m^6^A MTases (37,41,47) and RNA m^6^_2_A MTases (39,46,48), conserved residues in the motif X helix make hydrophobic interactions that stabilize the structure, whereas residues in the preceding loop interact with the cofactor. In none of these other structures does the motif X region undergo a large-scale conformational change on binding of cofactor and substrate. In several cases, there are smaller adjustments, e.g. a 2 Å movement of residues in the motif X tail to achieve optimal interactions with DNA substrate and AdoMet in *Eco*Dam (49).

The RlmJ structure in complex with AdoMet shows that motif X closing can, but is not forced to, happen in absence of substrate. Binding of the substrate analogue AMP induces further contraction of the catalytic site. We propose an induced fit model where cofactor binding allows motif X to go to the closed conformation where it induces further conformational changes in the active site and in the HS. Thereby, motif X closing contributes to formation of a positively charged RNA-binding surface (Figure 4E) as well as to positioning of residues in the active site. Most likely, binding of the full-length RNA substrate will induce further conformational changes including a movement of D164 and K18 toward the N6 atom of the target base. Similar induced fit structural rearrangement on target binding has been observed in the DNA C-MTase M.*Hha*I (50). After methylation and dissociation of the substrate from RlmJ, the motif X tail has to open to release AdoHcy and bind AdoMet before the next reaction. In T4Dam, a functionally similar movement of the helix covering the cofactor has been suggested (51).

### Substrate RNA recognition by RlmJ

The specificity of rRNA and tRNA MTases is in general based on the recognition of tertiary structure (52) in combination with an active site that correctly positions a particular target base. We have shown that RlmJ specifically methylates A2030 of *in vitro*-transcribed 23S rRNA. Thus, the substrate of RlmJ does not need any prior modifications. Furthermore, RlmJ site specifically modifies A2030 of the H72 fragment (Figure 1A) with similar efficiency as full-length 23S rRNA, showing that the major recognition elements reside within this fragment. This suggests that RlmJ can methylate A2030 before the 23S RNA is completely transcribed, processed and folded, consistent with the modification occurring early in ribosome biogenesis (4). Most likely, RlmJ recognizes a combination of a short A-form helix and some part of the eight unpaired nucleotides of the loop. In the mature ribosome, this loop is inaccessible and involved in tertiary interactions with the 5′ end of 23S domain II and with the PTC region of domain V (Figure 1B). The inability of RlmJ to modify single-stranded DNA with the corresponding sequence may be explained by, for example, specificity for an A-type double helix.

The presence of a dedicated RlmJ enzyme in *E. coli* shows that there at some growth condition must be an evolutionary pressure for transfer of a single methyl group to the N6 atom of A2030 in 23S RNA. Further details of the RlmJ-catalyzed methylation will be investigated in future experiments.

## ACCESSION NUMBERS

PDB accession numbers: 4BLU, 4BLV, 4BLW.

## SUPPLEMENTARY DATA

Supplementary Data are available at NAR Online.

## FUNDING

Swedish Research Council [project grants and URRC Linneaus center to A.C.F. and M.S.]; Swedish Foundation for Strategic Research, KAW (RiboCORE); Magnus Bergvall Foundation (to M.S.); Wenner-Gren Foundation Postdoctoral Fellowship (to T.R.S.). Funding for open access charge: Swedish Research Council.

*Conflict of interest statement*. None declared.

## Supplementary Material

Supplementary Data
