# Supplementary material for: Structural and functional insights into the molecular mechanism of rRNA m6A methyltransferase RlmJ
Source: Nucleic Acids Res. 2013 Aug 13;41(20):9537–48. doi: 10.1093/nar/gkt719 (PMC3814359; doi:10.1093/nar/gkt719)
Supplement: Supplementary Data [file supp_41_20_9537__index.html]

Structural and functional insights into the molecular mechanism of rRNA m6A methyltransferase RlmJ — Structural and functional insights into the molecular mechanism of rRNA m6A methyltransferase RlmJ — Supplementary Data 

# Structural and functional insights into the molecular mechanism of rRNA m6A methyltransferase RlmJ

## Supplementary Data

files

**Files in this Data Supplement:**

- Supplementary Data - pdf file
