# Supplementary material for: Structural and functional insights into the molecular mechanism of rRNA m6A methyltransferase RlmJ
Source: Nucleic Acids Res. 2013 Aug 13;41(20):9537–48. doi: 10.1093/nar/gkt719 (PMC3814359; doi:10.1093/nar/gkt719)
Supplement: Supplementary Data [file supp_gkt719_nar-01385-h-2013-File008.pdf]

Supporting Online Material for

# Crystal structure of RlmJ, the m$^6$A methyltransferase of A2030 in *Escherichia coli* 23S rRNA

Avinash S. Punekar, Josefine Liljeruhm, Tyson R. Shepherd, Anthony C. Forster and Maria Selmer*

* To whom correspondance should be addressed:

maria.selmer@icm.uu.se

This pdf includes:

Tables S1 and S2

Figures S1, S2, S3 and S4

**Table S1**. Oligonucleotides

| **Oligonucleotides used for preparation of RNA and DNA substrates** | |
|---|---|
| H72 Fwd | TAATACGACTCACTATAGGGAA |
| WT H72 Rev | GTACACTGCATCTTCACAGCGAGTTCCCTATAGTGAGTCGTATTA |
| A2030C Rev | GTACACTGCATCT**G**CACAGCGAGTTCCCTATAGTGAGTCGTATTA |
| A2030G Rev | GTACACTGCATCT**C**CACAGCGAGTTCCCTATAGTGAGTCGTATTA |
| A2030U Rev | GTACACTGCATCT**A**CACAGCGAGTTCCCTATAGTGAGTCGTATTA |
| ssDNA Fwd | GAACTCGCTGTGAAGATGCAGTGTAC |

| **Oligonucleotides used for preparation of RlmJ mutants** | |
|---|---|
| D164A Fwd | GCCGTGGTTTAATCCTTATCG**CT**CCGCCGTATGAAATGAAAAC |
| D164A Rev | GTTTTCATTTCATACGGCGG**AG**CGATAAGGATTAAACCACGGC |
| H6D Fwd | CTTATGCTCAGTTATCGC**G**A**T**AGCTTTCACGCTGGCAAC |
| H6D Rev | GTTGCCAGCGTGAAAG**C**T**A**TCGCGATAACTGAGCATAAG |
| Y4A Fwd | ATACCCTTATGCTCAGT**GC**TCGCCACAGCTTTCACGC |
| Y4A Rev | GCGTGAAAGCTGTGG**CG**AGCACTGAGCATAAGGGTAT |
| Y4F Fwd | ATACCCTTATGCTCAGT**TTC**CGCCACAGCTTTCACGC |
| Y4F Rev | GCGTGAAAGCTGTGGCG**GAA**ACTGAGCATAAGGGTAT |
| K18A Fwd | CACGCCGACGTCCTT**GC**ACATACCGTTCAGAGC |
| K18A Rev | GCTCTGAACGGTATGT**GC**AAGGACGTCGGCGTG |
| K18R Fwd | CACGCCGACGTCCTT**CGT**CATACCGTTCAGAGC |
| K18R Rev | GCTCTGAACGGTATG**ACG**AAGGACGTCGGCGTG |

**Table S2**. Results from DALI (31) search with $RlmJ_{APO}$.

| Match No. | PDB entry | Protein | Z-score | RMSD | Aligned residues | Seq. Id. (%) | Organism |
|---|---|---|---|---|---|---|---|
| 1 | 2OO3 | LPL1258, protein involved in catabolism of external DNA | 30.4 | 1.6 | 216 | 37 | *Legionella pneumophila* |
| 2 | 2H00 | MTase-like protein 16 | 14.2 | 3.2 | 168 | 14 | *Homo sapiens* |
| 3 | 4AX8 | WbdD, a bifunctional kinase/MTase | 13.9 | 3.3 | 176 | 11 | *Escherichia coli* |
| 4 | 1T43 | Release factor glutamine MTase HemK / PrmC | 13.9 | 2.7 | 163 | 13 | *Escherichia coli* |
| 5 | 1VID | Catechol O-MTase | 13.8 | 2.7 | 158 | 11 | *Rattus norvegicus* |
| 6 | 3TR6 | O-MTase | 13.7 | 2.8 | 158 | 15 | *Coxiella burnetii* |
| 7 | 4GEK | tRNA (cmo5U34) MTase | 13.7 | 2.7 | 163 | 10 | *Escherichia coli* |
| 8 | 3TMA | TrmN, tRNA $m^2$G6 (N2-methylguanosine) MTase | 13.5 | 2.7 | 159 | 16 | *Thermus thermophilus* |
| 9 | 3GRZ | Ribosomal protein L11 MTase | 13.2 | 2.6 | 150 | 12 | *Lactobacillus delbrueckii* |
| 10 | 3P9N | RsmD, 16S rRNA $m^2$G966 (N2-methylguanosine) MTase | 13.1 | 2.7 | 151 | 15 | *Mycobacterium tuberculosis* |
| 11 | 3LHD | TrmI, tRNA (adenine(57)-N(1)/adenine(58)-N(1)) MTase | 13.1 | 3.3 | 157 | 13 | *Pyrococcus abyssi* |
| 12 | 3V8V | RlmKL, 23S rRNA m7G2069 and m2G2445 MTase | 13.0 | 2.8 | 159 | 14 | *Escherichia coli* |
| 13 | 3K0B | Predicted N6-adenine-specific DNA MTase | 12.9 | 3.0 | 157 | 11 | *Listeria monocytogenes* |
| 14 | 3C0K | RlmI, 23S rRNA m5C1962 MTase | 12.6 | 3.3 | 169 | 11 | *Escherichia coli* |
| 15 | 2PJD | RsmC, 16S rRNA m2G1207 MTase | 12.4 | 2.9 | 155 | 14 | *Escherichia coli* |
| 16 | 3TKA | RsmH, 16S rRNA m(4)C1402 MTase | 12.3 | 2.9 | 159 | 11 | *Escherichia coli* |
| 17 | 4AUK | RlmM, 23S rRNA (cytidine2498-2'-O) MTase | 12.0 | 2.7 | 143 | 10 | *Escherichia coli* |
| 18 | 3Q87 | N6-adenine specific DNA MTase | 11.9 | 2.5 | 141 | 13 | *Encephalitozoon cuniculi* |
| 19 | 3FTC | RsmA / KsgA, 16S rRNA $m^6_2$A (N6-dimethyl A1518 and A1519) MTase | 11.9 | 3.0 | 151 | 13 | *Aquifex aeolicus* |
| 20 | 2DPM | DpnM, N6 adenine specific DNA modification MTase | 11.9 | 2.5 | 148 | 7 | *Streptococcus pneumoniae* |
| 21 | 1QAO | ErmC', 16S rRNA $m^6_2$A (N6-dimethyl A2085) MTase | 11.8 | 3.3 | 152 | 14 | *Bacillus subtilis* |
| 22 | 3FUT | RsmA / KsgA, 16S rRNA $m^6_2$A (N6-dimethyl A1518 and A1519) MTase | 11.2 | 3.3 | 150 | 9 | *Thermus thermophilus* |

| Match No. | PDB entry | Protein | Z-score | RMSD | Aligned residues | Seq. Id. (%) | Organism |
|---|---|---|---|---|---|---|---|
| 23 | 1QYR | RsmA / KsgA, 16S rRNA $m^6{}_2A$ (N6-dimethyl A1518 and A1519) MTase | 11.1 | 3.3 | 151 | 9 | *Escherichia coli* |
| 24 | 3LKD | Type I restriction-modification MTase subunit | 10.8 | 3.3 | 179 | 16 | *Streptococcus thermophilus* |
| 25 | 1G38 | M.TaqI, N6 adenine specific DNA modification MTase | 10.8 | 3.4 | 158 | 15 | *Thermus aquaticus* |
| 26 | 3G8A | RsmG, 16S rRNA m7G MTase | 10.7 | 3.4 | 156 | 11 | *Thermus thermophilus* |
| 27 | 3GRU | RsmA / KsgA / Dim1, 16S rRNA $m^6{}_2A$ (N6-dimethyl A1518 and A1519) MTase | 10.6 | 3.1 | 152 | 13 | *Methanocaldococcus jannaschii* |
| 28 | 1P91 | RlmA, 23S rRNA m1G745 MTase | 10.5 | 3.3 | 152 | 13 | *Escherichia coli* |
| 29 | 2OKC | Type I restriction enzyme StySJI M protein | 10.4 | 3.2 | 169 | 14 | *Bacteroides thetaiotaomicron* |
| 30 | 2ORE | EcoDam, N6 adenine specific DNA MTase | 10.3 | 3.0 | 147 | 10 | *Escherichia coli* |
| 31 | 1YFL | T4Dam, N6 adenine specific DNA MTase | 10.2 | 3.4 | 147 | 9 | *Enterobacteria phage T4* |
| 32 | 3S1S | RM.BpuSI, type IIG restriction endonuclease | 10.1 | 3.6 | 179 | 9 | *Bacillus pumilus* |
| 33 | 1DCT | M.HaeIII, C5 cytosine specific DNA modification MTase | 9.5 | 3.4 | 149 | 12 | *Haemophilus influenzae* |
| 34 | 3PT6 | Dnmt1, DNA (cytosine-5)-MTase 1 | 7.8 | 3.9 | 168 | 13 | *Mus musculus* |

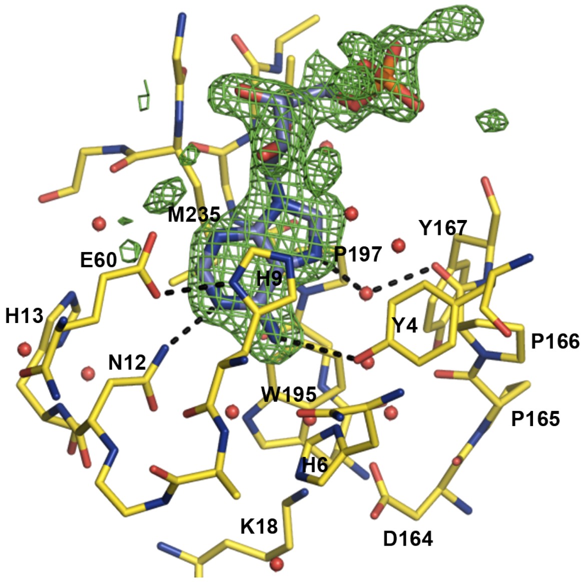


**Figure S1**. The substrate-binding site in RlmJ. The un-biased $F_o$-$F_c$ omit map (green) of AMP is contoured at 2.2 σ (0.3503 $e^-/Å^3$). AMP is in blue, the interacting residues within hydrogen bonding distance are shown in yellow, waters are shown as red spheres and hydrogen bonds as dotted lines.

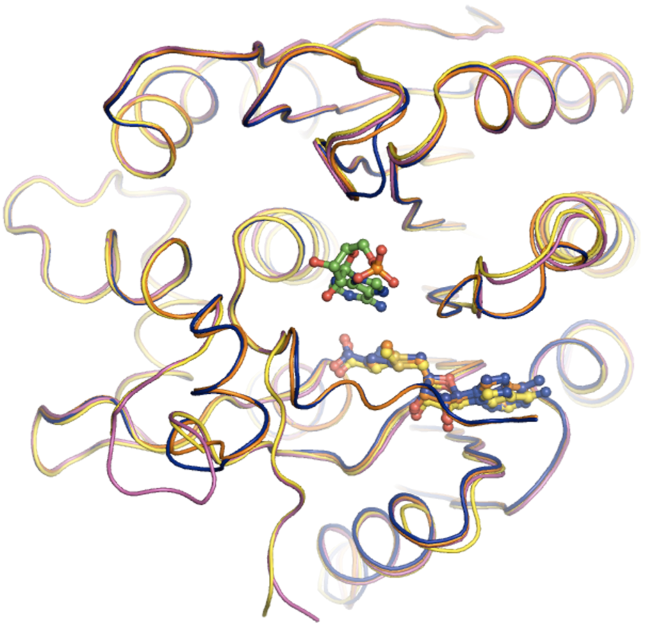

**Figure S2**. Constriction of the cofactor-binding pocket and the substrate-binding pocket due to the rearrangement of the N-terminal motif X tail and loop regions of motifs II, IV and VIII bring the substrate and cofactor closer to each other for catalysis. $RlmJ_{APO}$ (pink), $RlmJ_{SAM}$ molecule A (yellow), $RlmJ_{SAM}$ molecule B (orange) and $RlmJ_{SAH\text{-}AMP}$ (blue). AdoMet is shown in yellow and orange, AdoHcy is shown in blue and AMP is shown in green.

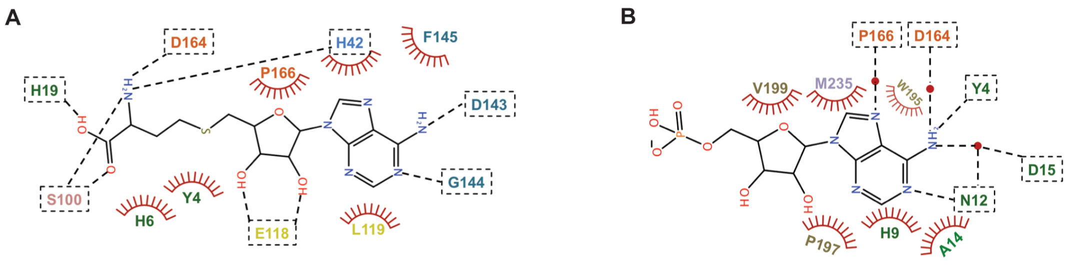


**Figure S3**. Schematic representation of the cofactor (AdoHcy) binding site (**A**) and the substrate (AMP) binding site (**B**). Residues are colored according to the different motifs: I (light blue), II (yellow), III (cyan), IV (orange), VI (olive), VIII (violet) and X (green). Hydrogen bond interactions are shown as dotted lines. Waters are shown as red dots. Residues in dotted box make hydrogen bond interactions and residues shown with red arcs make hydrophobic contacts.

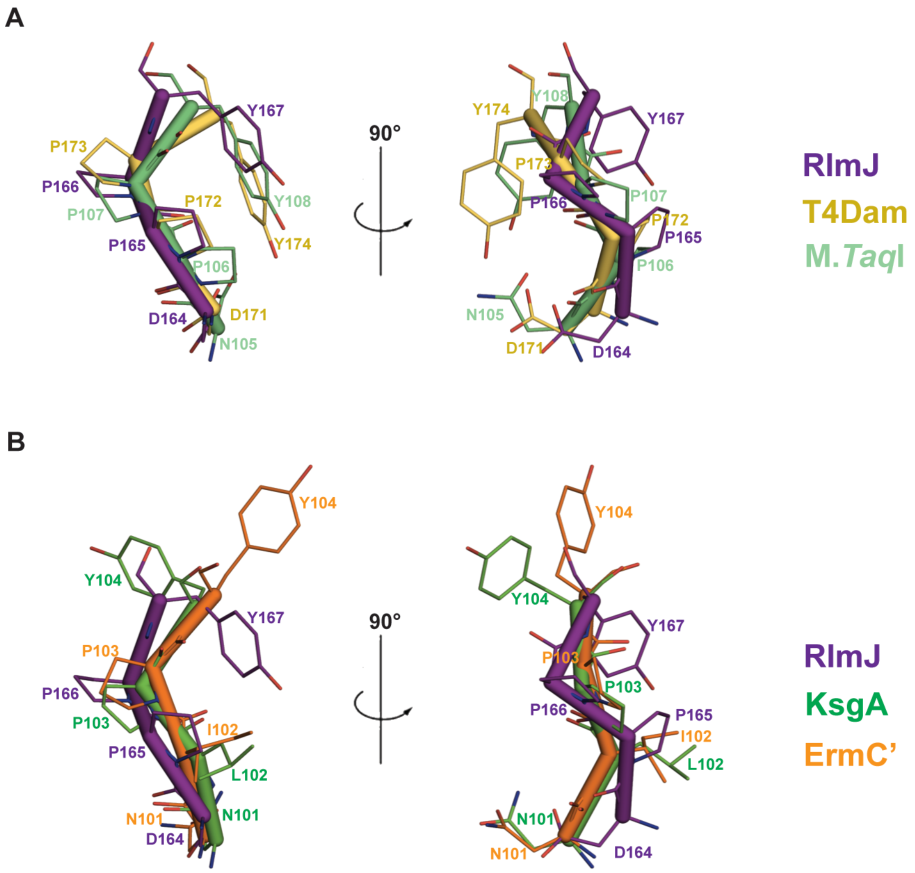


**Figure S4**. Structural comparison of the catalytic motif IV residues in $\text{RlmJ}_{\text{SAH-AMP}}$ ($_{164}$DPPY$_{167}$, purple) with (**A**) m$^6$A DNA MTases T4Dam (PDB 1YFL (43), $_{171}$DPPY$_{174}$, golden yellow) and M.*Taq*I (PDB 1G38 (39), $_{105}$NPPY$_{108}$, pale green); and (**B**) m$^6_2$A RNA MTases KsgA (PDB 3FTF (46) $_{101}$NLPY$_{104}$, dark green) and ErmC' (PDB 1QAM (41), $_{101}$NIPY$_{104}$, orange).
